# Pricing risk-based catastrophe bonds for earthquakes at an urban scale

**DOI:** 10.1038/s41598-022-13588-1

**Published:** 2022-06-13

**Authors:** Harsh K. Mistry, Domenico Lombardi

**Affiliations:** 1 Department of Mechanical, Aerospace and Civil Engineering, University of Manchester, Manchester, UK; 2 Centre for Crisis Studies and Mitigation, University of Manchester, Manchester, UK

**Keywords:** Natural hazards, Civil engineering

## Abstract

Catastrophe risk-based bonds are used by governments, financial institutions and (re)insurers to transfer the financial risk associated to the occurrence of catastrophic events, such as earthquakes, to the capital market. In this study, we show how municipalities prone to earthquakes can use this type of insurance-linked security to protect their building stock and communities from economic losses, and ultimately increase their earthquake resilience. We consider Benevento, a middle-sized historical town in southern Italy, as a case study, although the same approach is applicable to other urban areas in seismically active regions. One of the crucial steps in pricing catastrophe bonds is the computation of aggregate losses. We compute direct economic losses for each exposed asset based on high spatial resolution hazard and exposure models. Finally, we use the simulated loss data to price two types of catastrophe bonds (zero-coupon and coupon bonds) for different thresholds and maturity times. Although the present application focuses on earthquakes, the framework can potentially be applied to other natural disasters, such as hurricanes, floods, and other extreme weather events.

## Introduction

**Figure 1:**
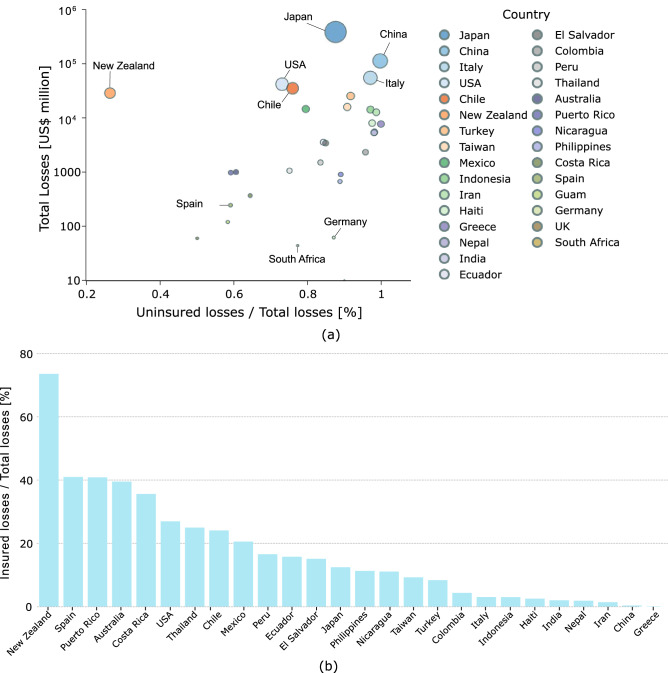
(**a**) Country-wise distribution of total losses vs. ratio of uninsured losses to total losses^2^; (**b**) earthquake insurance penetration for different countries located in seismically active regions. The ratio is defined as insured losses to total losses and is plotted in percent^2^.

Earthquakes are major natural disasters that can inflict severe and long-lasting socio-economic consequences on the affected communities. Differently from other natural disasters, such as hurricanes, volcanic eruptions and floods, which can be forecasted and/or monitored enough in advance to evacuate and thus protect the impacted communities, earthquakes are sudden and unpredictable events that can cause human and socio-economic losses over large urban areas before any mitigation interventions can be effectively deployed. Recent earthquakes such as the 2011 Tohoku Earthquake have shown that as seismic-prone regions become more urbanised and developed, economic and human losses can be high even in countries like Japan with well-established earthquake preparedness and seismic prevention programmes. With an estimated total economic loss in excess of $200 billion, the 2011 Tohoku Earthquake is the costliest earthquake in history. Chen et al.^1^ estimated comparable economic losses for a potential 7.8 magnitude earthquake striking Southern California. As shown by the distribution of insured and uninsured economic losses in Fig. 1a, adverse economic consequences induced by earthquake remain widespread, with countries such as Chile, China, Greece, Italy, Turkey and USA particularly affected. While human losses can be largely reduced by adopting earthquake-resistant design procedures and retrofitting existing buildings, the burden of economic losses can be mitigated through a number of different financial protection instruments that can transfer part of the financial risk to (re)insurance companies or the capital market. The most common financial instruments used to hedge against financial losses caused by future seismic events include: earthquake insurance and insurance-linked securities. In an earthquake insurance policy, a share of the economic risk is transferred from the buildings’ owners to the insurer in exchange of regular payments (i.e., premiums). The insurer can then transfer all, or part of, the risk to a reinsurance company through alternate risk transfer (ART) products. Compared to traditional buildings insurance, the use of earthquake insurance remains infrequent even in countries prone to strong earthquakes. As shown in Fig. 1a,b, New Zealand is a notable exception with over 75$$\%$$ of the total earthquake losses insured. Other countries such as China, Greece, India, Italy, Iran and Japan present a relatively low insurance penetration despite their long history of damaging earthquakes.

Catastrophe risk bonds (CAT bonds in short) are a type of insurance-linked financial security that transfers the financial risks of future natural catastrophes to the capital markets. In exchange, investors, such as hedge funds, pension funds, and other institutional investors, receive competitive interest rates over the duration of the bond, typically between 3 and 5 years. In the event of a natural disaster triggering the payout, the bond holders may lose a part or the entire principal depending on the economic losses incurred; if no triggering events take place at maturity, the investors receive their initial investment (i.e., principal) in addition to the regular yield returns.

The main challenge in deploying CAT bonds at scale lies in the complexity in assessing the risk of a heterogeneous building stock subjected to rare events. There are only a few studies in the literature that have dealt with CAT bonds. Burnecki and Kukla^3^ computed the non-arbitrage prices for a zero-coupon and coupon CAT bond using a doubly stochastic Poisson process based and 10 years catastrophe loss data. Hardle and Cabrera^4^ studied real parametric catastrophe bonds for earthquakes in Mexico, and concluded that the use of reinsurance and CAT bond provides lower exposure to default than the use of reinsurance alone. They calibrated the CAT bond using a hybrid (modelled-index) type of trigger mechanism that combined modelled losses and magnitude of the seismic events. Ma and Ma^5^ introduced a bond pricing approach with stochastic interest rates and a compound nonhomogeneous Poisson process for losses. They used catastrophe loss data from 1985 to 2010 to calibrate the Poisson process and loss parameters of their model. Shao et al.^6^ introduced formulas for pricing CAT bonds using a stochastic interest rate and aggregate losses obtained from a nonhomogeneous Poisson process and semi-Markov process. Shao et al.^7^ focused on pricing of nuclear catastrophe bonds using a Markov-dependent environment based on the Cox-Ingersoll-Ross (CIR) and Vasichek interest rate models. They also studied the influence of using different interest rate models on the zero-coupon bond pricing and concluded that although the CIR model is considered superior over the Vasichek model due to its ability to provide non-zero interest rate values, the difference in the bond pricing is not significant. Hofer et al.^8^ introduced a formula for pricing CAT bonds for different accepted levels of risk, taking into account the role of uncertainty in the claim arrival and severity processes. Hofer et al.^9^ proposed a framework for designing CAT bonds for a spatially distributed portfolio. They implemented the proposed framework on a national level, considering a portfolio of assets comprising of all residential buildings in Italy. To simplify the analysis, they considered a single value of PGA for each municipality, thus assuming that all assets in a given municipality were located at the centroid of the municipality. Furthermore, the economic losses were computed based on single replacement cost value for all assets.

The present work builds on the pioneering works by Hofer et al.^8,9^, and goes beyond by making an attempt to incorporate a high spatial resolution for the hazard and exposure models. The motivation behind this work is to increase the accuracy of the loss estimates, which is crucial for making the catastrophe bond a more attractive tool for risk management strategies that public entities can put in place to mitigate the risk of future catastrophe losses. More specifically, we develop a high spatial resolution hazard model that has the advantage of considering the effect of local site conditions on the seismic hazard estimates. The importance of local site effects on the seismic hazard estimates has been recognised and discussed in several studies and may result in significant amplification of the ground shaking in the presence of soft deposits^10–12^. Additionally, we explicitly account for the epistemic uncertainty in the hazard model by considering two different source models^13^: (1) area source model; (2) fault source and background model. The seismic hazard results computed by each model are then combined within a logic tree. The final seismic hazard model is computed by generating a stochastic synthetic earthquake catalogue that provides a peak ground acceleration (PGA) estimates for each individual asset within the study area, whose values take into account possible ground shaking amplification due to local site effects. Results show that the seismic hazard can vary significantly even within a given municipality, thus affecting the distribution of losses. We develop also a high spatial resolution exposure model, in which replacement costs are determined from data available from the Italian National Institute of Statistics^14^; the replacement costs for each asset are estimated taking into account the type of asset (i.e., commercial, productive, residential and tertiary) and location of asset within the municipality (i.e., old town, central urban area, semi-central urban area, semi-central area and agriculture area see Fig. 4a for more details). We finally convolute the hazard, exposure and vulnerability models to obtain the aggregate losses used for pricing zero-coupon and coupon bonds for different maturity times and thresholds. The proposed framework is illustrated by considering a case study that consists of typical medium-size city in a seismically active area in southern Italy. It should be mentioned from the outset that, although earthquakes are responsible for multi-seismic hazards including landslides, tsunami, liquefaction, surface rupture, and even nuclear disasters as seen in the 2011 Tohoku Earthquake, the current work assumes that earthquake-induced ground shaking is the main hazard contributing to the seismic risk and consequent financial loss.

## Study area

**Figure 2:**
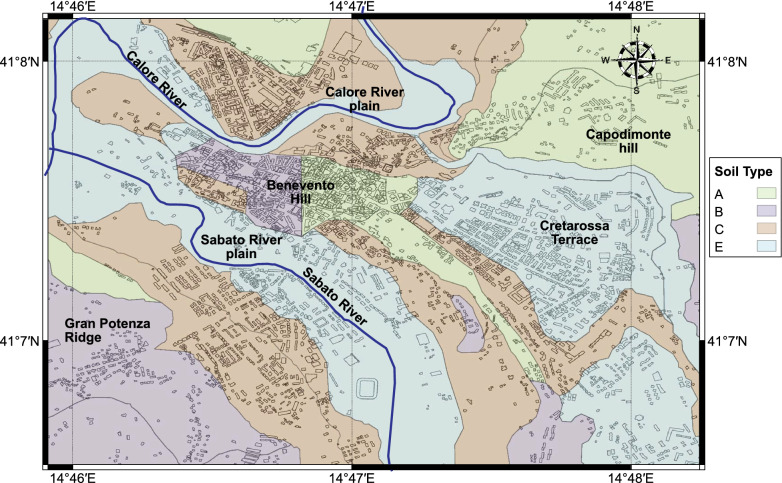
Geological map of Benevento, including ground classification according to Eurocode 8^17^. The average shear wave velocity $V_{s,30}$; for each ground type ranges as follows: (**A**) (Rock, $V_{s,30}$ > 800 m/s-in green); (**B**) (very dense sand and gravel, $V_{s,30}$: 360–800 m/s-in purple); (**C**) (dense sand and gravel, $V_{s,30}$: 180–360 m/s -in brown); (**E**) (surface alluvium layer with thickness between 5 and 20 m, $V_{s,30}$: 180–360 m/s or <180 m/s-in light blue). This figure is created with QGIS version 3.18.2 (https://www.qgis.org/).

The city of Benevento is a typical example of a middle-sized historical Italian town. It has a population of approximately 60,000 inhabitants and a heterogeneous building stock comprising of masonry and reinforced concrete buildings, spanning from 1 to 6 storeys. The city is located in the Campania region (southern Italy), about 50 km northeast of Naples. The seismicity of the study area is dominated by the Sannio-Matese seismogenic structure which has been responsible for large historical earthquakes between 1456 and 1805, with five events with intensity $> X$ of the Mercalli-Cancani-Sieberg (MCS) scale. Since 1805, the study area has experienced few moderate earthquakes and seismic swarms with magnitude < 3^15^. The shallow geology of the city is complex and characterised by a rapid variation of geological units (see Fig. 2); these include: the Gran Potenza ridge, the Sabato and Calore river plains, the Benevento hill, the Capodimonte hill and the Creatarossa terrace^16^. Shallow soft layers made of fillings, colluvial soils and recent alluvia ($V_s$ between 200 and 300 m/s) are found in the the Sabato and Calore river plains; colluvial deposits are present along the slopes that encircle the Benevento hill. Stiff cemented conglomerates ($V_s$ between 1000 and 1730 m/s) outcrop at the crest of the Benevento hill. The Capodimonte hill includes stiff weathered conglomerates deposits ($V_s > 800$ m/s). In the Cretarossa terrace, the conglomerates are overlaid by fluvio-lacustrine deposits from the Lower-Middle Pleistocene ($V_S<270$ m/s). Stiff clays and silts ($V_s$ between 600 and 800 m/s) over layers of sandstone and conglomerates form the Gran Potenza ridge. The sharp contrast in shear wave velocities of the different geological units makes the area prone to local site effects, with important implications on the spatial distribution and severity of the seismic hazard and losses as documented in the “Results” section.

## Results

### Seismic hazard model

**Figure 3:**
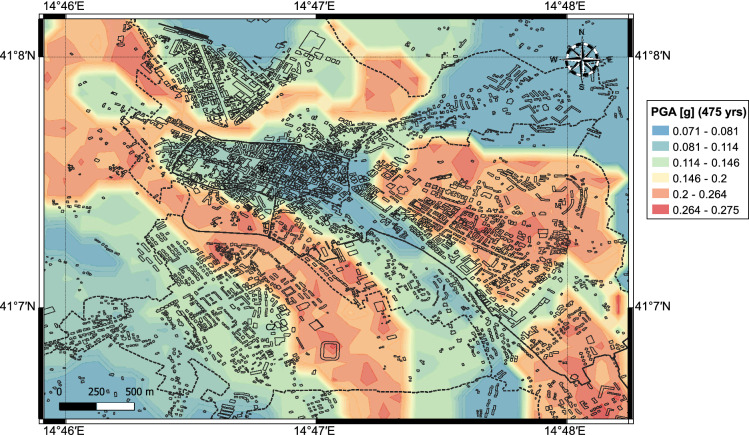
Seismic Hazard model: results express peak ground acceleration values for return period of 475 years (i.e., probability of exceedance of 10% in 50 years). This figure is created with QGIS version 3.18.2 (https://www.qgis.org/).

We considered an area between 41$^{\circ }$65$^{\prime }$–41$^{\circ }$82$^{\prime }$ N and 14$^{\circ }$46$^{\prime }$–14$^{\circ }$48$^{\prime }$ E of latitude and longitude. Figure 3 shows the spatial distribution of the seismic hazard in terms of peak ground acceleration (PGA) for a probability of exceedance of 10$\%$ in 50 years (475 years return period) with a 150m $\times$ 150m grid spacing. It can be seen that the PGA distribution varies between 0.071 and 0.275 g, depending on the stiffness and layering of the different geological units. More specifically, the lowest PGA estimates (<0.081 g) are found at the eastern extremity of the old town, where the stiff conglomerates outcrop the north-northwest trending ridge that forms the Benevento hill. The PGA increases up to 0.146 g along the sides of the ridge, in correspondence of the colluvial deposits that encircle the hill. PGA estimates <0.114 g are computed for the Capodimonte hill. The highest PGA estimates (up to 0.275 g) are found at the Cretarossa terrace unit, where the presence of soft young fluvio-lacustrine deposits results in a significant amplification of the ground motion. High PGA (between 0.200 g and 0.275 g) are computed for the Sabato and Calore river plains made by soft and young deposits of colluvial and alluvium soils.

### Exposure model

**Figure 4:**
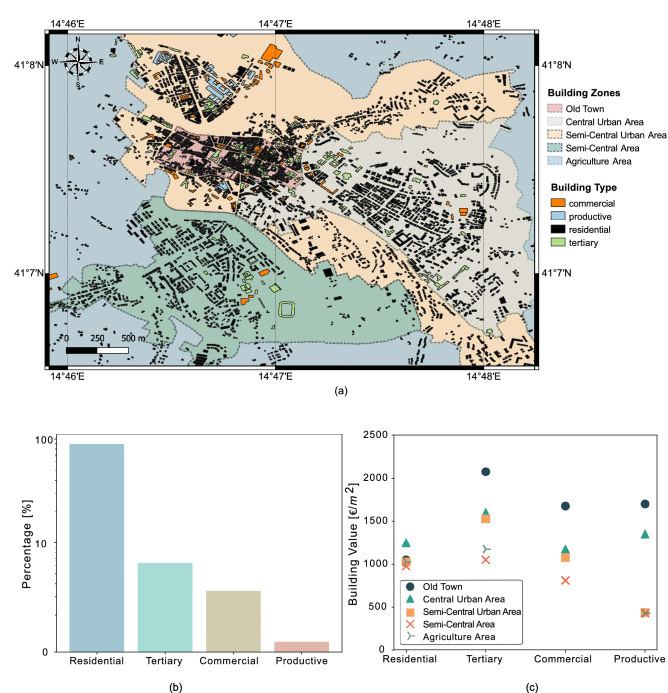
Exposure model: (**a**) building footprints of the Benevento region classified in four categories: commercial (orange); productive (blue); residential (black); tertiary (green); Territorial zones categorised as per Italian Revenue Agency^18^: (1) old town (pink); (2) central urban area (off white); (3) semi-central urban area (ivory); (4) semi-central area (dark green); (5) agriculture area (blue); This figure is created with QGIS version 3.18.2 (https://www.qgis.org/); (**b**) proportion of buildings in each category in the Benevento region: residential (blue); tertiary (green); commercial (yellow); productive (red); (**c**) per square meter value of each building typology based on different territorial zones: old town (circle); central urban area (triangle); semi-central urban area (cross); agriculture area (tri right).

We build a high spatial resolution exposure model for the city of Benevento (see Fig. 4). Firstly, we divide the urban area into five homogeneous territorial zones, referred to as *OMI* by the Italian Revenue Agency^18^, namely: old town (B1), central urban area (B2), semi-central urban area (C1), semi-central (C2) and agricultural area (D1). The old town (B1) is located along the north-northwest trending ridge of the Benevento hill, and its building stock comprises of masonry houses. The central urban area (B2) extends over the Cretarossa terrace; this is the most densely populated area of the study region as it comprises the majority of the multi-storey reinforced concrete buildings. The semi-central urban (C1) and semi-central areas (C2) extend over the Capodimonte hill and the Sabato river plain, and their building stock is dominated by masonry buildings up to 2–3 storey. Within each territorial zone, we classify building types based on their use into: *commercial*, *productive*, *residential* and *tertiary*. We then assign real estate values based on the latest datasets available from the Italian National Institute of Statistics^14^. The building footprints are extracted from the map database OpenStreetMap^19^ and Google Earth Engine^20^. From the compiled exposure model, it can be seen that the majority of buildings is residential, with a high density of reinforced concrete buildings in the central urban area.

### Vulnerability and loss model

**Figure 5:**
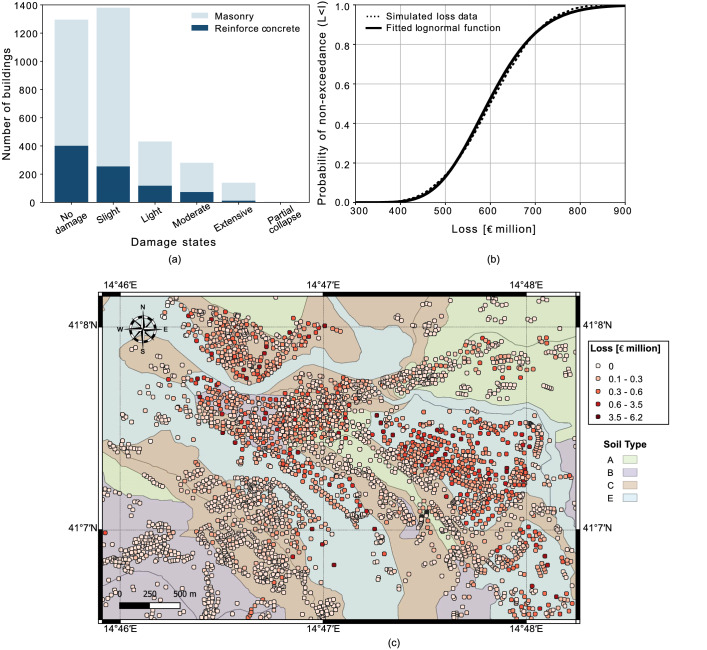
Loss results for vulnerability analysis: (**a**) proportions of masonry (light blue) and reinforce concrete (dark blue) building in each damage states; (**b**) simulated non-exceedance loss curve (dotted black line). The results shown are the mean of 10000 Monte Carlo simulations. Fitting of lognormal cumulative distribution function (solid black line) on the simulated loss data; (**c**) spatially distributed losses (mean of 10000 simulations) for the portfolio of buildings. Geological units: $V_{s,30}$ for each ground type ranges as follows: A (Rock, $V_{s,30}$ > 800 m/s-in green); B (very dense sand and gravel, $V_{s,30}$: 360–800 m/s-in purple); C (dense sand and gravel, $V_{s,30}$: 180–360 m/s-in brown); E (surface alluvium layer with thickness between 5 and 20 m, $V_{s,30}$: 180–360 m/s or < 180 m/s-in light blue). This figure is created with QGIS version 3.18.2 (https://www.qgis.org/).

The main output of vulnerability and loss models includes the non-exceedance probability of losses and the spatial distribution of mean losses for each building considered in the exposure model. Figure 5*a* highlights the proportion of each structure type (i.e., masonry and reinforce concrete) for different damage states. We observe that the majority of the buildings experience no damage or slight damage. The majority of reinforced concrete structures experience no damage while the majority of masonry structures experience slight damage. Figure 5*b* shows the non-exceedance probability of losses $P\left( L<l \right)$ for the entire building stock obtained from the simulations (dotted black line). The loss curve is one of the inputs for pricing the CAT bonds for the portfolio of interest. The spatial distribution of mean losses of 10000 simulations are presented in Fig. 5*c*. High level of losses are observed in the Cretarossa terrace region and Benevento hill which ranges from 0.3 to 6.5 €million, while for Capodimonte hill and Gran Potenza ridge lower loss levels (i.e., 0.1–0.3 €million) are observed.

### Risk-based catastrophe bond pricing

The intensity parameter defining the homogeneous Poisson process ($\lambda _M = 0.252$) is calibrated based on the temporal occurrence of events within the seismic source zone where the study region is located; this is source zone 313 in Fig. 8. The recurrence data is obtained from the historical earthquake catalogue compiled during the SHARE project^13^. The parameters defining the loss process are usually calibrated based on historical catastrophe loss and claim data^3–7^. However, owing to the paucity of historical loss data for the study region, there is a need for a risk-based simulation to predict the impact of seismic events on the portfolio of assets. In the present study, the loss process is defined in terms of mean ($\mu = 6.387$) and standard deviation ($\sigma = 0.153$), whose values have been calibrated by fitting a lognormal cumulative distribution function to the simulated loss data (see Fig. 5*b*).

**Figure 6:**
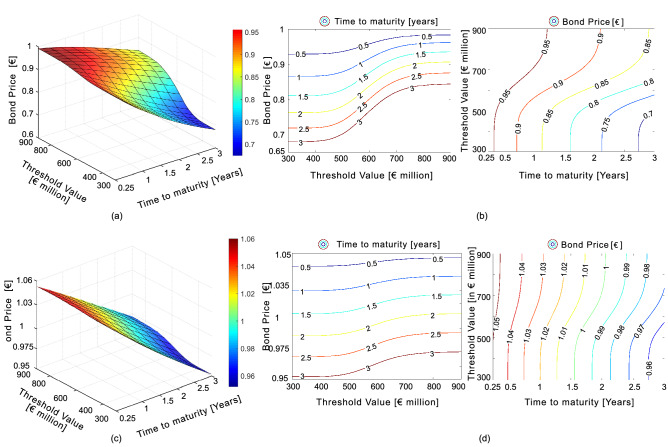
Zero-coupon CAT bond pricing: (**a**) surface plot of bond price for various $T-D$ levels; (**b**) left: contour plot of threshold versus bond price (each contour line refers to different time to maturity levels); right: contour plot of time to maturity versus threshold value (each contour line refers to different bond price levels); Coupon CAT bond pricing: (**c**) surface plot of bond price for various $T-D$ levels; (**d**) left: contour plot of threshold versus bond price (each contour line refers to different time to maturity levels); right: contour plot of time to maturity versus threshold value (each contour line refers to different bond price levels).

Figure 6a,c shows the valuation for zero-coupon and coupon CAT bond for varied time to maturity and threshold levels, respectively. In case of zero-coupon bond (see Fig. 6a), we observe that the price of bond decreases with increasing time to maturity. At a given maturity *T*, the bond price increases with increasing threshold level. Similar trends can be observed for coupon CAT bonds (see Fig. 6c). In comparison with zero-coupon CAT bonds, the price for coupon CAT bonds are higher for the chosen maturity time and threshold level combinations. Additionally, in the case of Coupon CAT bonds, the probability of receiving more coupon payments increases as the time elapses. Therefore coupon bonds are more beneficial to investors as they yield higher prices while protecting their principal amount. Figure 6b (left),d (left) show the variation of bond price with respect to threshold value for the zero-coupon and coupon CAT bond, where each line refers to different time to maturity. Figure 6b (right),d (right) illustrate the bond price lines for varied levels of time to maturity and threshold value.

## Methods

**Figure 7:**
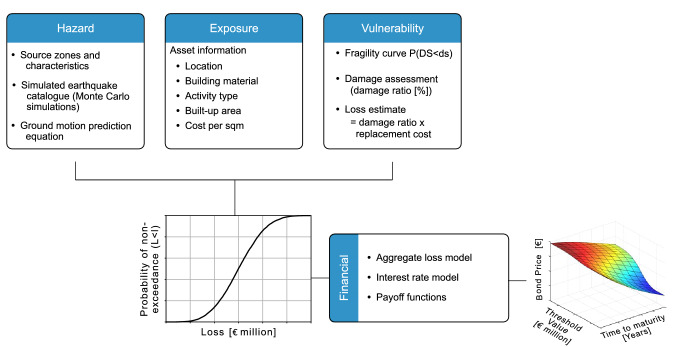
Methodology flow chart for pricing of risk-based catastrophe bonds: hazard, exposure, vulnerability and financial module.

The methodology for pricing of risk-based catastrophe bonds used in the present work can be divided into four main models: hazard, exposure, vulnerability and financial models (see Fig. 7). The hazard model is defined based on the results from a probabilistic seismic hazard analysis (PSHA) that takes into account the local site condition at the location of each asset (see also “Study area” section). More specifically, we adopt a simulation-based approach using the Monte Carlo method to perform a stochastic PSHA and produce a set of synthetic earthquake events. The results are expressed in terms of a spatial distribution of PGA for a 475 years return period. The exposure model provides details on the exposed assets, including asset geographical coordinates, construction material type, economic activity type, built-up area, and real estate building value (per square metre). The vulnerability model includes two components: (1) fragility function and (2) loss function. The former defines the probability of exceeding a damage state conditioned on the intensity measure, while the latter defines the probability distribution of loss for respective damage state. The main outcome of vulnerability module is the non-exceedance loss function which is obtained by fitting a lognormal distribution to simulated loss data. Further, this non-exceedance loss function is used as an input for computing a region-specific aggregate loss. In the present work, the target loss is defined in terms of replacement costs incurred by the damaged assets. These costs are computed from the real estate building values publicly available from the Italian National Institute of Statistics^14^ for the different building types (i.e., commercial, productive, residential and tertiary) and zones where the damaged asset is located. For the municipality of Benevento five different zones are considered, namely: old town, central urban area, semi-central urban area, semi-central area and agriculture area see Fig. 4a for more details. It is worth noting that the present exposure model ignores additional indirect economic losses (also known as consequential losses) caused by loss of revenues due to potential business interruptions; the indirect losses are more difficult to estimate from the available data, especially for a heterogeneous portfolio of assets such that considered in this study. Finally, since we are considering assets located within the municipality of Benevento, without loss of generality, we can assume that the hypothetical CAT bonds computed in this study are issued by a public authority, such as the local council. The financial module is the final step where the aggregate losses, interest rate model and payoff functions are used to price zero-coupon and coupon catastrophe bonds for different loss thresholds and times to maturity. Further details of each model are presented in the sections below.

### Hazard model

The seismic hazard defines the ground-motion intensity expected at the site in a given time period. This is often expressed in terms of a spatial distribution of intensity measure parameter, such as a PGA, or spectral accelerations SA at different vibration periods. We use a standard PSHA to determine the ground-shaking hazard, expressed in terms of PGA; this involves the following steps^21,22^:

- Definition of earthquake source model,
- Definition of earthquake recurrence relationship,
- Definition of ground-motion models

**Figure 8:**
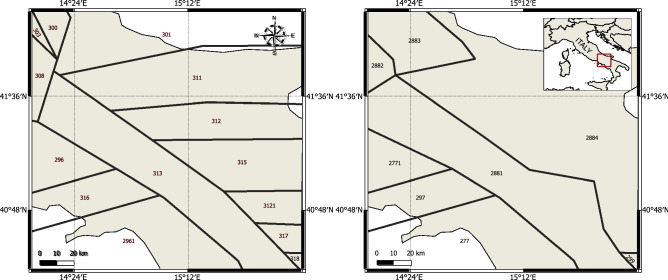
Seismic source models used for the assessment of the seismic hazard of the study region; left: area source model; right: fault source and background model;^13^. This figure is created with QGIS version 3.18.2 (https://www.qgis.org/).

For the definition of the earthquake source model, we use the two models developed during the *Seismic Hazard Harmonization in Europe* (SHARE) project^13^, namely: (1) area source model (see Fig. 8:left); (2) fault source and background model (see Figure 8:right). The results computed from each source model are combined together using a logic tree, where equal weights are assigned to each model. We adopt a homogeneous Poisson process to describe the temporal occurrence of earthquakes within each source zone, where the occurrence time between each event, *t*, also known as inter-arrival time, is computed by^23^:

1 $$\begin{aligned} t=-\frac{\ln (1-u)}{\lambda _{M}} \end{aligned}$$

where *u* is a random number between 0 and 1; $\lambda _{M}$ is the annual occurrence rate for a specific zone which can be defined by the truncated Gutenberg-Richter relationship:

2 $$\begin{aligned} \lambda _{M}= & {} N_0 \frac{e^{-\beta M_{min}}-e^{-\beta M_{max}}}{1-e^{-\beta M_{max}}}\end{aligned}$$

3 $$\begin{aligned} N_{0}= & {} 10^{a} \end{aligned}$$

4 $$\begin{aligned} \beta= & {} \log _{10}(b) \end{aligned}$$

where *a* and *b* are the Gutenberg-Richter parameters estimated from historical earthquake catalogue; $M_{min}$ and $M_{max}$ denote the minimum and maximum magnitude respectively. For each seismic event, the location of the epicentre is derived from a uniform probability distribution whereas the magnitude of each event is computed based on the Gutenberg-Richter relationship given by:

5 $$\begin{aligned} m=-\frac{\ln (e^{-\beta M_{min}} - (1-u) \times [e^{-\beta M_{min}} - e^{-\beta M_{max}}] )}{\beta } \end{aligned}$$

All the recurrence parameters and minimum and maximum magnitudes for each source zone are taken from the SHARE project^13^.

We use a simulation-based approach^23,24^ to generate a synthetic earthquake catalogue with a large number of stochastic events that are temporally and spatially complete. This catalogue contains information such as the time of occurrence, location, focal depth and the intensity for each event. Once the synthetic catalogue is generated, we calculate the PGA using the ground motion prediction equation (GMPE) proposed by Bindi et al.^25^.

Finally, we compute the annual rate of exceeding a given PGA value as follows:

6 $$\begin{aligned} v_{GM}(\ge gm) = \sum _{model,i=1}^{m}w_{i} \sum _{zone,j=1}^{q} \left[ \lambda _{M,ij} \iint _{\Omega m,r,ij} P(GM\ge gm|m,r) f_{M,R,ij}(m,r) \,dm\,dr \right] \end{aligned}$$

where $v_{GM}$ refers to the annual occurrence rate of exceeding a specific value of ground motion intensity *gm*; $w_i$ is the weight for the *i*th model, where i ranges from 1 to m, with m being the total number of source models; j is the total number of source zones defined in each source model, which ranges from 1 to n (total number of source zones in a source model); $\lambda _{M,ij}$ denotes the annual rate of occurrence of earthquake events corresponding to *j*th zone of the *i*th model; $P(GM \ge gm|m,r)$ is the probability that a ground motion intensity exceeds a specific ground motion intensity value, conditioned on the magnitude *m* and distance *r*; $f_{M,R,ij}$ is the probability density function for $M-R$ combination; $\Omega _{m,r,ij}$ refers to the sample space for *m*, *r* combination.

### Vulnerability model

We build a vulnerability model based on a suite of fragility and loss curves. A fragility curve provides the probability of a exceeding a specific damage state conditioned on the intensity measure. The loss functions provide the probability distribution of loss (in terms of mean loss ratio and standard deviation) for each damage state. We consider the following five damage states expressed in terms of damage ratios (in percent)^26^: DS1 (Slight) = 0–10%; DS2 (Light) = 10–40%; DS3 (Moderate) = 40–70%; DS4 (Extensive) = 70–90%; DS5 (Partial Collapse) = 90–100%. We adopt fragility curves by Rota et al.^27^ for masonry structures and Rosti et al.^28^ for reinforced structures. We estimate the economic losses as the product of damage ratio and the real estate building value provided by the exposure model. Further these simulated losses are used to calibrate the loss parameters of the aggregate loss model which are required for the CAT bond pricing discussed in the subsequent section.

### Catastrophe risk bond pricing

We adopt the Cox-Ingersoll-Ross model^29^ for the computation of the spot interest rate for different maturity times and use the non-exceedance loss curve from the vulnerability model to compute the aggregate losses. We price CAT bonds with a face value of €1 at time $t=0$ years considering two types of payoff functions: zero-coupon and coupon. The coupon *C* is taken as €0.6. The parameters defining the Cox-Ingersoll-Ross (CIR) model ($k=0.0984$, $\theta =2.04\%$, $\sigma =4.77\%$, $\lambda _{r}=-0.01$, $r_{0}=2.04\%$) are calibrated based on the 3-month maturity U.S. monthly treasury bill data for the period 1994–2013^6^. The time to maturity *T* is taken as $T \in \left[ 0.25,3 \right]$ years, while the threshold loss *D* is taken in the range: *D* $\in$ [€300*m*, €900*m*].

#### Interest rate model

The Cox-Ingersoll-Ross (CIR) model is based on the assumption that the spot interest rate follows a mean-reverting square-root process, mathematically expressed by

7 $$\begin{aligned} dr\left( t\right) =k\left[ \theta -r\left( t\right) \right] dt + \sigma \sqrt{r\left( t\right) }\,dW\left( t\right) \end{aligned}$$

where *k*, $\theta$, $\sigma$ are the positive constants representing the mean-reverting force measurements, long-run mean of the interest rate and volatility parameter for the interest rate, respectively; $W\left( t\right)$ denotes the standard Wiener process for t$\in$[0,T], $\left\{ W\left( t\right) :t\in [0,T]\right\}$. The non-negative interest rates are ensured by using the condition $2k\theta >\sigma ^{2}$. The pure-discount T-bond at time t can be estimated as^29^:

8 $$\begin{aligned} B_{CIR}\left( t,T\right)= & {} A\left( t,T\right) e^{-B\left( t,T\right) r\left( t\right) } \end{aligned}$$

9 $$\begin{aligned} A\left( t,T\right)= & {} \left[ \dfrac{2\gamma e^{\left( k+\lambda _{r}+h\right) \left( T-t\right) /2}}{2\gamma +\left( k+\lambda _{r}+\gamma \right) \left( e^{\left( T-t\right) h}-1\right) }\right] ^{2k\theta /{\sigma ^2}} \end{aligned}$$

10 $$\begin{aligned} B\left( t,T\right)= & {} \left[ \dfrac{2\left( e^{\left( T-t\right) \gamma }-1\right) }{2\gamma +\left( k+\lambda _{r}+\gamma \right) \left( e^{\left( T-t\right) \gamma }-1\right) }\right] \end{aligned}$$

11 $$\begin{aligned} \gamma= & {} \sqrt{\left( k+\lambda _{r}\right) ^{2}+2\sigma ^{2}} \end{aligned}$$

where, $\lambda _{r}\left( t\right)$ represents the market risks; $\left( T-t\right)$ is the time to maturity. The main advantage of using the CIR model over models is that it prevents interest rates to be negative.

#### Aggregate loss model

The aggregate losses are estimated based on the compound distribution of two processes: (1) catastrophe events process, $N\left( t\right)$; (2) catastrophe severity process, $X_{n}$. The former process defines the frequency of occurrence of catastrophic events, while the latter determines the severity of losses incurred during an event. For the computation of aggregate losses, we adopt the approach proposed by Ma and Ma^5^, which is based on three assumptions:

1. The flow of potential catastrophic events of certain magnitude for a region covered in the contract can be described by a Poisson point process, N$\left( t\right)$ $\left( t\in \left[ 0,T\right] \right)$, defined by the intensity parameter $\lambda _{M}$. The time instants, $t_{i}$ of the potential catastrophic loss events are denoted by $0\le t_{1} \le \cdots \le t_{n} \le \cdots \le T$.
2. The time when the aggregate losses $\left( L_{t}\right)$ exceed a certain threshold level $\left( D\right)$ is defined as $\tau =inf\left\{ t:L\left( t\right) \ge D\right\}$; this defines the occurrence of the triggering event.
3. The severity of catastrophic events in each time instant $\left( t_{n}\right)$ is an independent and identically distributed $\left( i.i.d\right)$ random variable, $\left\{ X_n\right\} _{n=1,...}$. The cumulative distribution function of this random variable is given by $F\left( x\right) =P\left( X_{n} < x\right)$.

The aggregate losses is computed as

12 $$\begin{aligned} L\left( t\right) =\sum _{n=1}^{N\left( t\right) }X_{n} \end{aligned}$$

where $N\left( t\right)$ and $X_{n}$ refers to the frequency of catastrophic events and severity of loss, respectively.

#### Payoff function

The payoff function defines the amount to be paid on maturity if no trigger event ($L_{T}>D$) occurs and the amount to be paid as payoff in case of occurrence of a triggering event. We use two payoff functions for pricing zero-coupon and coupon-type bonds. The payoff function $\left( P_{ZC}\right)$ for a simple zero-coupon CAT bond, where a fraction of face value is to be paid to the bondholders on the occurrence of triggering event, is defined according to^5^:

13 $$\begin{aligned} P_{ZC}= {\left\{ \begin{array}{ll} Z, &{} L\left( T\right) \le D\\ \eta Z, &{} L\left( T\right) > D \end{array}\right. } \end{aligned}$$

where, *Z* is the maturity value (face value) that is paid to the bond holders if no triggering event occurs until maturity, *T*; $\eta$ is the fraction of face value to be paid to bond holders on the occurrence of a triggering event until maturity; $L\left( T\right)$ and *D* represent the aggregated loss at maturity time *T* and the threshold (or trigger) value, respectively. In presence of a coupon CAT bond and no triggering event, the bond holders receives the face value *Z* at the maturity time in addition to the coupon payments *C* paid annually (or at specific intervals). On the other hand, if a triggering event occurs, the coupon payment terminates before maturity time, with the bondholders receiving the face value of the bond. The payoff function for a coupon CAT bond $P_{C}$ is given by^5^ by:

14 $$\begin{aligned} P_{C}= {\left\{ \begin{array}{ll} Z+C, &{} L\left( T\right) \le D\\ Z, &{} L\left( T\right) > D \end{array}\right. } \end{aligned}$$

Finally, the pricing of zero-coupon and coupon CAT bonds is carried out by combining the outcomes of the interest rate model, aggregate loss model and the payoff function. Mathematically, the price of zero-coupon ($V_{zc}$) and coupon-type ($V_{c}$) CAT bonds at a given time *t*, assuming a risk-neutralized pricing measure *Q*, is given by^5^:

15 $$\begin{aligned} V_{zc}\left( t\right)= & {} E^{Q} \left( Ze^{-\int _{t}^{T}r_{s}ds} P_{ZC}|F_{t} \right) =B_{CIR}\left( t,T\right) Z\left\{ \left[ F\left( D,T\right) +\eta \left[ 1-F\left( D,T\right) \right] \right] \right\} \end{aligned}$$

16 $$\begin{aligned} V_{c}\left( t\right)= & {} E^{Q} \left( Ze^{-\int _{t}^{T}r_{s}ds} P_{C}|F_{t} \right) =B_{CIR}\left( t,T\right) \left[ Z + C F\left( D,T\right) \right] \end{aligned}$$

17 $$\begin{aligned} F\left( D,T\right)= & {} \sum _{n=0}^{\infty } e^{-\lambda _{M}T}\dfrac{(\lambda _{M}T)^{n}}{n!}F^{n}(D) \end{aligned}$$

where, $B_{CIR}$ is the spot interest rate at time *t* (see “Catastrophe risk bond pricing 'section); *Z* denotes the maturity value; $\left( T-t\right)$ is the time remaining to maturity; *F*(*D*, *T*) is the probability that aggregate losses are less than the threshold loss $P\left[ X_{1}+X_{2}+\cdots +X_{n}\le D\right]$; $\lambda _{M}$ refers to the intensity of the Poisson point process at time instant *t*; $F^{n}(D)$ is the n-th convolution of *F*.

## Conclusion

Earthquake-prone countries can suffer from severe economic losses in the event of strong earthquakes, which can hinder their economic growth and exacerbate inequalities. Developments in probabilistic seismic risk assessments, and better computational capabilities, provide an opportunity to quantify the potential economic losses that future earthquakes can inflict on urban areas with high confidence and high spatial resolution. This is a crucial step for increasing the appetite of investors to bear financial risk of catastrophe losses and for public entities to consider risk financing instruments as a valid risk management strategy to transfer part of the financial risk arising from catastrophes to the capital market. In the present work, we develop high resolution hazard and exposure models for a middle-sized historical town in southern Italy, and compute future catastrophic economic losses considering the effect of local site condition on the distribution of the hazard, and up-to-date real estate information on the exposed assets. We then use the computed economic losses to determine aggregate losses and price two types of catastrophe bonds. Differently from reinsurance, CAT bonds provide a more secure financial instrument that national or local governments can use to transfer the financial risk of catastrophes to the capital market. On the other hand, investors can take on the risk in return for an attractive yield while investing their funds in a diversified portfolio that makes a positive contribution towards the Sustainable Development Goals (SDGs) set out by the United Nations.
